# Role of gonadotropin-releasing hormone 2 and its receptor in human reproductive cancers

**DOI:** 10.3389/fendo.2023.1341162

**Published:** 2024-01-08

**Authors:** Amy T. Desaulniers, Brett R. White

**Affiliations:** ^1^ School of Veterinary Medicine and Biomedical Sciences, University of Nebraska-Lincoln, Lincoln, NE, United States; ^2^ Department of Animal Science, University of Nebraska-Lincoln, Lincoln, NE, United States

**Keywords:** GnRH2, GnRHR2, reproductive cancer, breast, prostate, endometrial, ovarian, placental

## Abstract

Gonadotropin-releasing hormone (GnRH1) and its receptor (GnRHR1) drive reproduction by regulating gonadotropins. Another form, GnRH2, and its receptor (GnRHR2), also exist in mammals. In humans, *GnRH2* and *GnRHR2* genes are present, but coding errors in the *GnRHR2* gene are predicted to hinder full-length protein production. Nonetheless, mounting evidence supports the presence of a functional GnRHR2 in humans. GnRH2 and its receptor have been identified throughout the body, including peripheral reproductive tissues like the ovary, uterus, breast, and prostate. In addition, GnRH2 and its receptor have been detected in a wide number of reproductive cancer cells in humans. Notably, GnRH2 analogues have potent anti-proliferative, pro-apoptotic, and/or anti-metastatic effects on various reproductive cancers, including endometrial, breast, placental, ovarian, and prostate. Thus, GnRH2 is an emerging target to treat human reproductive cancers.

## Introduction

### GnRH2

Hypothalamic GnRH1 binds GnRHR1 on gonadotropes, promoting gonadotropin [luteinizing hormone (LH) and follicle-stimulating hormone (FSH)] synthesis/secretion. Another form, GnRH2, is also present in mammals (1). GnRH2 is ubiquitously expressed (1) and originates from the *GnRH2* gene (chromosome 20) in humans (2). Both decapeptides, GnRH2 and GnRH1 have a 70% sequence identity (3). Amino acid substitutions in GnRH2 enhance its stability (4) and half-life (5, 6) compared with GnRH1.

### GnRHR2

A 7-transmembrane (TM) G-protein coupled receptor (GPCR) specific to GnRHR2 is present in mammals and ubiquitously expressed (1). GnRH2 binds its cognate receptor with greater affinity than GnRHR1 (24-fold increase (7)), leading to greater activity (up to 440-fold increase (8–10)). In contrast, GnRH1 exhibits 12-fold greater activity at GnRHR1 compared to GnRH2 (9, 11). Thus, GnRHR2 displays greater selectivity for GnRH2, whereas GnRHR1 binds/activates both decapeptides reasonably well (8). Both receptors utilize G_αq/11_ to trigger IP_3_ synthesis and activate protein kinase C (PKC (8, 12, 13)), but downstream signaling pathways diverge (7). Additionally, GnRH2 activation of GnRHR1 initiates different signaling pathways than GnRH1 (14), suggesting that GnRH2 elicits different physiological effects than GnRH1 at GnRHR1.

Humans maintain a full-length *GnRHR2* gene (chromosome 1 (10)), although a frameshift mutation and premature stop codon are predicted to prevent full-length receptor production (15, 16). Nevertheless, there is mounting evidence for a functional GnRHR2 in humans (12, 17–23), potentially via production of a 5-TM GnRHR2 (17). Mammals, including humans, produce functional 5-TM GPCRs (11, 24–26). Notably, pigs produce 5-TM GnRHR2 transcripts with translatable protein characteristics, resulting from alternative splicing and an alternative start codon (27, 28). In addition to the full-length *GnRHR2* gene (chromosome 1), a truncated *GnRHR2* gene (chromosome 14) is also present in humans (10), which is more transcriptionally active and widely expressed (29).

## GnRH2 and GnRHR2 in human reproductive cancers

The tumor microenvironment is dependent on unchecked cell division, cytokines, anti-apoptotic mediators, and immune cell recruitment, which are controlled by a variety of important biomolecules like hormones, growth factors, cytokines, and immune mediators (e.g., Toll-like receptors) (30–32). For decades, we have known that GnRH1 and GnRHR1 are expressed in reproductive tumors and GnRH1 analogues inhibit cancer cell proliferation (19, 33–38). In fact, the newest approaches utilize GnRH1-tagged nanoparticles to directly target chemotherapeutics into cancer cells (39). In addition, elevated expression of *GnRH1* and *GnRHR1* in bladder cancer is linked with better survival in men but worse survival in women, suggesting possible regulation of the GnRH1/GnRHR1 system by gonadal steroids in non-reproductive tissues (40). Recently, GnRH2 and GnRHR2 have been detected in reproductive cancer cells (**Table 1**). Like GnRH1, GnRH2 analogues inhibit cancer cell proliferation; however, GnRH2 is often more potent (18, 19, 21, 44, 57). It remains unclear if GnRHR2 or GnRHR1 are mediating these effects. A role for GnRHR2 seems plausible due to difficulty detecting high affinity receptors for GnRH1 in peripheral reproductive tissues (34). Also, high concentrations of GnRH1 analogues are required to suppress cancer cell proliferation (35). Indeed, many independent groups have reported evidence for a functional GnRHR2 in reproductive cancer cells (12, 18, 19, 21, 44, 58). Moreover, new data suggests that differential methylation of *GnRH2* may affect cancer progression in non-reproductive organs (59, 60). Likewise, gene polymorphisms are increasingly being linked with the onset of cancer (31). *GnRH2* gene polymorphisms have been linked with bone cancer (61, 62). Thus, GnRH2 and GnRHR2 may be novel, unexploited cancer targets.

**Table 1:** Presence of GnRH2 and/or GnRHR2 in malignant reproductive tissues and cells of humans.

Tissue or Cell line	Origin	GnRH2^a^	GnRHR2^a^	References
Female				
Tissues				
Breast	Adenocarcinoma	+	+	(41–43)
Endometrium (uterus)	Adenocarcinoma	+	+	(44, 45)
Ovarian	Carcinoma	+	+	(44, 46)
Cancer cell lines				
MDAMB-231	Breast adenocarcinoma	+		(41)
MCF-7	Breast adenocarcinoma	+	+	(41, 43, 47)
T47D	Breast carcinoma		+	(43)
HeLa	Cervical adenocarcinoma		+	(9, 22)
HEC-1A	Endometrial adenocarcinoma		+	(19)
HEC-1B	Endometrial adenocarcinoma		+	(18)
Ishikawa	Endometrial adenocarcinoma		+	(19)
HHUA	Endometrial adenocarcinoma		+	(48)
A2780	Ovarian carcinoma	+		(49)
EFO-21	Ovarian cystadenocarcinoma		+	(19)
EFO-27	Ovarian adenocarcinoma		–	(18)
OVCAR-3	Ovarian adenocarcinoma	+	+	(19, 49, 50)
SK-OV-3	Ovarian adenocarcinoma	+	+	(19, 49, 50)
CaOV-3	Ovarian adenocarcinoma	+		(49, 50)
BG-1	Ovarian adenocarcinoma		+	(18, 49)
JEG-3	Placental carcinoma	+		(51–53)
Male				
Tissues				
Prostate	Adenocarcinoma	+	+	(44, 54, 55)
Cancer cell lines				
ALVA-41	Prostate adenocarcinoma		+	(22)
PPC-1	Prostate adenocarcinoma		+	(22)
DU-145	Prostate carcinoma	+	+	(22, 48, 56)
LNCaP	Prostate adenocarcinoma	+	+	(55, 56)
PC3	Prostate adenocarcinoma	+	+	(55, 56)

a The presence of a (+) symbol indicates that either mRNA or protein has been identified whereas a (-) symbol specifies that the tissue was negative. Blanks designate cell lines that have not yet been examined.

### Breast

In 2002, Chen et al. (41) demonstrated that *GnRH2* mRNA was overexpressed in cancerous versus normal breast tissue. In another study, *GnRH2* expression was 2-fold greater in malignant compared to normal tissue (42). Moreover, *GnRH2* expression in breast cancer samples correlated with indices of a poorer prognosis (42). GnRHR2 immunostaining is detectable in breast cancer cells (**Table 1** (43)), suggesting autocrine/paracrine interactions. Others identified direct anti-proliferative effects of GnRH2 on breast cancer cells (47, 63). In MCF-7 and T47D cells, GnRH2 agonist pre-treatment interrupted epidermal growth factor (EGF) signaling, ablating EGF-mediated autophosphorylation of EGF receptor and the induction of the mitogen-activated protein kinase (MAPK), extracellular-signal-regulated kinase 1/2 (ERK1/2 (43)). Furthermore, a GnRH2 agonist reversed 4OH-tamoxifen insensitivity of breast cancer cells (43). In MCF-7 cells, GnRH2 downregulated proteins required for translation and cell proliferation (41).

In addition to reducing cell proliferation, GnRH2 analogues induce apoptosis. GnRH2 antagonists stimulated loss of mitochondrial membrane potential and apoptosis in breast cancer cells via p38 MAPK and c-Jun N-terminal kinase (JNK) pathways, culminating in activation of the pro-apoptotic protein, BAX (47). The same antagonists failed to activate protein kinase B (also known as AKT) or ERK1/2 (47). In a different study, GnRH2 antagonists induced apoptosis in triple negative MDA-MB-231 breast cancer cells [lack estrogen receptors, progesterone receptors, and human EGF receptor 2 (HER2)], which was mediated by p38 MAPK signaling, loss of mitochondrial membrane potential, and capsase-3 activation (23). *GnRHR1* knockdown failed to fully ablate GnRH2 antagonist-mediated apoptosis, implicating GnRHR2 (23). Moreover, GnRH2 antagonists completely inhibited breast cancer tumor growth in nude mice (23).

Notably, MCF-7 cells take up fluorescently labeled GnRH2-conjugates effectively, which is under investigation as a method of targeted drug delivery (64). Indeed, GnRH2 analogues conjugated to cytotoxic drugs (e.g., daunorubicin) have shown promising anti-tumor effects *in vitro* (64, 65). In addition to anti-proliferative and pro-apoptotic effects, GnRH2 analogues also have anti-metastatic properties. For example, breast cancer cell migration to bone and invasion of an artificial basement membrane were attenuated by GnRH2, although to a lesser extent than GnRH1 (66).

### Uterine

#### Endometrium

GnRH2 has been detected in endometrial carcinomas (45), whereas *GnRHR2* mRNA is present in both endometrial carcinomas (45) and endometrial cancer cells (HEC-1A, HEC-1B, HHUA, and Ishikawa (18, 19, 48; **Table 1**)). Further, GnRHR2 protein was identified in endometrial cells (44). Co-expression of GnRH2 and GnRHR2 suggest an autocrine/paracrine role in endometrial cancers (67). Several independent groups determined that GnRH2 analogues reduced endometrial cancer cell growth (18, 19, 21, 47, 57, 58, 68). GnRH2 more effectively inhibited the growth of HEC-1A and Ishikawa cells compared to the same dose of a potent GnRH1 agonist (triptorelin (18)). Triptorelin, cetrorelix (pan GnRHR antagonist), and GnRH2 exerted anti-proliferative effects on endometrial cancer cells (Ishikawa, HEC-1A, and HEC-1B) that produce *GnRHR1* and *GnRHR2* transcripts (18). *GnRHR1* knockdown ablated anti-proliferative effects of triptorelin but failed to ablate the efficacy of cetrorelix and GnRH2 (18).

The same group detected protein corresponding to the 5-TM GnRHR2 (43-kDA) in Ishikawa and HEC-1A cells (44). Their antibody was validated in part via detection of a 7-TM GnRHR2 band (54-kDa) in ovarian protein from marmoset monkeys (44), a species that produces a full-length GnRHR2 (9). Interestingly, radiolabeled GnRH2 binds a 43-kDA protein in human endometrial cancer cells (44). Both native GnRH2 and cetrorelix (pan GnRHR antagonist) were able to displace ^125^I-labeled GnRH2, but not triptorelin (GnRH1 agonist (44)). Of note, cetrorelix binds both GnRHR1 and GnRHR2 reasonably well (22, 69, 70), whereas triptorelin is highly specific for GnRHR1 (10). The authors hypothesized that these results occurred due to a functional 5-TM GnRHR2 (44). Indeed, both low and high affinity binding sites for GnRH1 were detectable in human endometrial cancer cells (71), implicating the presence of GnRHR1 (high) and GnRHR2 (low).

GnRH2 also has anti-proliferative effects in endometrial cancer cells. For example, a GnRH2 agonist attenuated the proliferative effects of growth factors on Ishikawa and HEC-1A cells (21). Specifically, the GnRH2 agonist activated phosphotyrosine phosphatase, which reduces the autophosphorylation of activated EGF receptors, and downregulated genes associated with EGF-mediated transcription, leading to reduced cell proliferation (21). Importantly, these effects persisted following *GnRHR1* deletion, implicating GnRHR2 (21). Furthermore, Park et al. (58) demonstrated reduced proliferation of HEC-1A cells after GnRH2 treatment, which was more effective than GnRH1.

The efficacy of GnRH analogues to induce apoptosis has been tested in endometrial cancer cell lines. Analogues of GnRH1 (agonist and antagonist) failed to induce apoptosis (57). In contrast, antagonists of GnRH2 induced apoptosis via caspase-3 activation, which appeared to be mediated by GnRHR1 (47). Likewise, GnRH2 induced apoptosis in Ishikawa cells via GnRHR1 (72). GnRH2 induced apoptosis and suppressed cell proliferation in endometrial carcinoma cell lines, with a greater effect observed in cells with PTEN knockdown (68). Additionally, GnRH2 reduced protein kinase B (AKT) and ERK1/2 activity in HEC-1A-ND cells (68). In animal models, growth of xenotransplants from HEC-1B cells in nude mice was suppressed by GnRH2 (57). Researchers have also found that a GnRH2 agonist enhanced cell migration through GnRHR1-mediated phosphorylation of ERK1/2 and JNK, leading to MAPK-dependent activation of matrix metalloproteinase-2 (MMP-2) in Ishikawa and ECC-1 cells (73). Notably, GnRH2 analogues have a more potent inhibitory effect than GnRH1 on proliferation of endometrial cancer cells (18, 19, 21, 44, 57). Interestingly, a metabolite of GnRH1 [GnRH-(1–5)] also regulates the progression of endometrial cancer (74); however, effects of GnRH2 metabolites on endometrial cancer cells have not yet been explored.

#### Myometrium

GnRH analogues are clinically utilized to treat leiomyomas, benign fibroids of the myometrium (75). Induction of a hypoestrogenic state is thought to drive fibroid involution (75). However, transcripts for both *GnRH2* and GnRHR2, as well as protein for GnRH2, were detected in normal myometrial tissue and leiomyomas of women, suggesting a direct effect of GnRH analogues on fibroid growth (76).

### Ovarian

Both GnRH2 and/or GnRHR2 have been detected in cancerous ovarian cells (**Table 1**). *GnRH2* was overexpressed in malignant compared to benign ovarian tumors or normal ovarian tissue (46). *GnRH2* expression in ovarian cancer cells appears to be mediated in part by gonadotropins. Choi et al. (49) reported that gonadotropin treatment (FSH or LH) reduced *GnRH2* expression in the majority of ovarian cancer cell lines tested (including OVCAR-3 cells); however, *GnRH1* mRNA was unaffected by treatment (49). *GnRHR1* mRNA was downregulated by FSH or LH in most ovarian cancer cell lines but *GnRHR2* expression was not examined (49). Converse to this data, Ling Soon et al. (51) found that a *GnRH2* promoter-luciferase reporter gene construct was activated by8-bromoadenosine-cAMP in OVCAR-3 cells (cAMP is a second messenger of LH and FSH (51)). The cause of the discrepancy has not been resolved in the literature but may relate to differences in treatment (LH/FSH versus 8-bromoadenosine-cAMP), dose, culture conditions, and/or testing of the endogenous cellular machinery versus luciferase assay. Notably, in post-menopausal women with ovarian tumors, there was a positive correlation between serum LH and FSH concentrations and *GnRH2* expression in ovarian tumor samples (46), suggesting a stimulatory role of the gonadotropins on GnRH2 expression *in vivo*. *GnRH2* expression is also regulated by EGF in ovarian cancer cells. For example, EGF upregulates *GnRH2* promoter activity in OVCAR-3 cells; an effect that is abolished in the presence of an EGF receptor inhibitor (77).

Due to its potent anti-proliferative effects, GnRH2 has garnered attention as a possible therapeutic for ovarian cancer treatment. Early studies demonstrated that treatment of both non-tumorigenic (IOSE-29) and tumorigenic (IOSE-29EC) cells with GnRH2 reduced cell proliferation (19, 50). Choi et al. (49) reported that GnRH2 agonists inhibited growth of ovarian cancer cells, an effect that was reversed by LH or FSH pre-treatment. Grundker et al. (19) showed that GnRH2 reduced ovarian cancer cell proliferation, outperforming equimolar triptorelin (a GnRH1 agonist) treatment. In SK-OV-3 ovarian cancer cells (expressing *GnRHR2* but not *GnRHR1*), GnRH2 exhibited powerful anti-proliferative effects, unlike triptorelin (19), suggesting that GnRHR2 is mediating these effects. However, others contested the assertion that *GnRHR1* is not expressed in SK-OV-3 cells (78), although they acknowledged that it may be present, but expressed at low levels (77). In another study, anti-proliferative effects of triptorelin were abolished after *GnRHR1* knockdown in ovarian cancer cells (EFO-21 and OVCAR-3), but effects of GnRH2 and the pan GnRHR antagonist, cetrorelix (binds both GnRHR1 and GnRHR2 (70)), persisted (18). Together, these findings suggest that GnRHR2 is functional in certain ovarian cancer cells. However, other groups provided evidence that GnRHR1 is involved in mediating anti-proliferative effects of GnRH2 (78). Thus, the exact receptor (GnRHR1 and/or GnRHR2) eliciting anti-proliferative effects of GnRH2 remains controversial.

Data from Eicke et al. (44) supports the presence of GnRHR2 protein in humans; immunostaining identified GnRHR2 in ovarian cancer samples. Both immunoblotting of protein and photo labeling studies of cell membrane fractions from ovarian cancer cells (EFO-21, SK-OV-3) resulted in a band corresponding to the 5-TM GnRHR2 isoform (43-kDa (44)). Competition experiments showed that triptorelin weakly competed for the binding site (43-kDa) compared to the stronger effect of cetrorelix (44) (pan GnRHR antagonist (70)). However, GnRH2 was the most potent competitor, indicating the presence of a functional 5-TM GnRHR2 in human ovarian cancer cells (44).

Subsequent studies have been conducted to determine the mechanism underlying anti-proliferative effects of GnRH2 on ovarian cancer cells. For example, GnRH2 treatment led to p38 MAPK activation, an effect which was reversed by SB203580 (p38 MAPK inhibitor (79)). Likewise, activator protein-1 was stimulated by GnRH2 but reduced in the presence of SB203580 (79). In OVCAR-3 cells, GnRH2 treatment inhibited cell growth, but this effect was abolished when cells were pre-treated with SB203580 (79). GnRH2 treatment also enhanced apoptosis, which was reversed with SB203580 pre-treatment (79). The same group showed that ERK1/2 (but not JNK) is involved in mediating anti-proliferative effects of GnRH2 (63). Others reported that GnRH2 mediated cell proliferation is dependent on PKC (78). In this study, however, data suggested that GnRHR1 (not GnRHR2) mediated these effects (78). Additional research demonstrated that GnRH2 treatment inhibited mitogenic effects of EGF in ovarian cancer cells (21). *GnRHR1* knockdown failed to prevent these effects, suggesting the contribution of GnRHR2.

Many researchers have investigated pro-apoptotic activities of GnRH2 analogues on ovarian cancer cells (47, 57, 79, 80). GnRH2 antagonists induced apoptosis by activating caspase-3 and effectively inhibited growth of human ovarian cancer xenotransplants in nude mice (57). Furthermore, GnRH2 antagonists activated p38 MAPK and JNK, resulting in activation of BAX mitochondrial dysfunction (loss of membrane potential, release of cytochrome C), and caspase-3 activation (47). Recent data also demonstrated that co-treatment of ovarian cancer cells with a glycolysis inhibitor and a GnRH2 antagonist reduced cell viability and increased apoptosis to a greater extent than each treatment individually (80).

The role of GnRH2 in ovarian cancer metastases has also been explored. Chen et al. (81) found that low doses of GnRH1 and GnRH2 promoted invasion of OVCAR-3 cells but had the opposite effect in SKOV-3 cells (both GnRH1 and GnRH2 inhibited invasion but only at high doses). *GnRHR1* knockdown abolished the effect of treatment in both cell types; however, *GnRHR2* expression was not examined (81). Treating SKOV-3 cells with either GnRH1 or GnRH2 led to reduced MMP-2 expression and increased secretion of tissue inhibitor of MMP-2 (TIMP2), both important mediators of ovarian carcinoma metastasis (81). Furthermore, GnRH1 and GnRH2 disrupted activation of the phosphatidylinositol-3-kinase (PI3K)/AKT pathway, which promotes proteolysis and invasion in ovarian cancer cells (81). Thus, in SKOV-3 cells, GnRH2 inhibits ovarian cancer invasion by regulating the balance of MMP2/TIMP2, and disrupting AKT-mediated proteolysis and invasion (81). In contrast, others reported that GnRH2 enhanced membrane type I metalloproteinase production via the PI3K/AKT pathway and phosphorylation of GSK3β in OVCAR-3 and CaOV-3 cells (82).

Chen et al. (81) hypothesized several different mechanisms that might enable two lines of ovarian cancer cells to exhibit different invasive responses to GnRH2 (presumably both via GnRHR1). Possible explanations include differences in inherent cell invasiveness and/or receptor expression levels, which is a known driver of differential cellular responses (83). For example, Chen et al. (81), found that low doses of GnRH2 promoted invasion in OVCAR-3 cells (with elevated GnRHR1 expression) unlike SKOV-3 cells (with low GnRHR1 expression). Interestingly, both SKOV-3 and OVCAR-3 cells express GnRHR2 (**Table 1**), but the level of expression has not been compared to our knowledge.

Interestingly, GnRH2 and EGF worked synergistically to promote invasion of OVCAR-3 and CaOV-3 cells, but not SKOV-3 cells (reduced endogenous *GnRHR1* expression (77)). *GnRHR1* knockdown in OVCAR-3 and CaOV-3 cells only partially inhibited invasiveness mediated by EGF (77), suggesting that GnRHR2 may be involved. Later studies demonstrated that EGF increased *GnRH2* expression in OVCAR-3 and CaOV-3 cells, potentially enhancing autocrine signaling (mediated by GnRHR1 (84)). Enhanced GnRHR1 signaling leads to increased production of the 37-kDa laminin receptor precursor, more tumor cell interactions with laminin in the extracellular matrix, and enhanced MMP-2 production (84). These data suggest that GnRH2 modulates pro- and anti-metastatic effects depending on the ovarian cancer cell type. This discrepancy has not yet been resolved but may be related to expression differences in GnRHR1 and/or GnRHR2 among cell types.

### Placental

*GnRH2* mRNA is present in the choriocarcinoma cell line, JEG-3 ((51–53); **Table 1**), and cAMP treatment activated the *GnRH2* promoter (51). Both GnRH1 and GnRH2 enhanced JEG-3 cell invasion (12) but *GnRHR1* knockdown only inhibited GnRH1-mediated effects, not GnRH2 (12), implicating GnRHR2. Furthermore, GnRH2 treatment of JEG-3 cells reduced cell proliferation, results which were ascribed to GnRHR1 (85). GnRHR2 has not been investigated in JEG-3 cells, although Eicke et al. (44) demonstrated evidence for a functional 5-TM GnRHR2 in human placentae.

### Prostate

GnRH1 agonists are commonly used to treat prostate cancer but increase the risk of adverse cardiovascular events (86), which highlights the need for more therapeutic options. In addition to normal tissue, GnRH2 is also present in hyperplastic and neoplastic prostate tissues ((54, 55); **Table 1**). Eicke et al. (44) reported immunoreactive GnRHR2 in prostate adenocarcinomas, specifically within epithelial (not stromal) cells. A recent study found an association between prostate cancer progression and a *GnRH2* gene polymorphism in Japanese men (87), although a separate study did not observe this link in Caucasian men (54). Therefore, GnRH2 and GnRHR2 may be involved in autocrine/paracrine regulation of prostate cancer progression.

Notably, *GnRH2* and *GnRHR2* are expressed in normal and cancerous prostate cell lines (22, 48, 55, 56). GnRH2 treatment reduced proliferation of all tested prostate cancer cell lines; these results were ascribed to GnRHR1 and activation of cAMP (56). Others showed that GnRH2 increased intracellular calcium levels via activation of the ryanodine receptor in androgen independent DU-145 cells (22). Likewise, a GnRH2-specific antagonist (trptorelix-1) induced cell death and prevented GnRH2-mediated calcium influx (22). Photoaffinity labeling suggested that GnRH2 binds with high affinity to a protein in prostate cancer cells (22), implicating GnRHR2.

Androgens enhanced *GnRH2* expression in prostate tumors by binding a putative androgen response element on the 5’ flanking region of the human *GnRH2* gene (55). Thus, anti-androgen therapy reduces *GnRH2* expression in tumor biopsies (55). Studies using a prostate xenograft model demonstrated that androgens enhanced *GnRH2* expression, whereas androgen deprivation reduced *GnRH2* expression (55). Consistent with this, *GnRH2* expression is elevated in prostate cancer cells (e.g., LNCaP cells) that produce androgen receptors (ARs) compared to those lacking ARs (e.g., PC3 cells (55)) and AR inhibition blocked androgen-mediated increases in *GnRH2* expression in LNCaP cells. Interestingly, GnRH2 treatment of LNCaP (AR positive) and PC3 (AR negative) cells led to reduced cell proliferation and migration, suggesting that these actions are not dependent on AR signaling (55).

The anti-proliferative activity of GnRH2 has garnered increasing attention as a therapeutic target. For example, Kim et al. (88) developed a GnRH2 specific antagonist, trptorelix-1, that effectively inhibited growth of PC3 cells *in vitro* and *ex vivo* (88). Moreover, trptorelix-1 decreased mitochondrial membrane potential and enhanced reactive oxygen species (ROS) within the cytoplasm and mitochondria (88). Antioxidant co-treatment partially protected against trptorelix-1-mediated growth inhibition. Furthermore, autophagosome formation was observed in the absence of apoptosis markers in prostate cancer cells treated with trptorelix-1, which induced cell signaling cascades consistent with autophagy (88).

The same group developed another GnRHR2 antagonist, SN09-2 (89). When compared to trptorelix-1, SN09-2 suppressed growth of prostate cancer cells, even at low concentrations, and was an effective inhibitor of PC3 xenograft growth. These effects were associated with mitochondrial accumulation of SN09-2, leading to mitochondrial dysfunction and ROS generation (89). Furthermore, SN09-2 induced markers of apoptosis in PC3 cells (89).

Researchers have also investigated the potential for targeted tumor treatment by incubating LNCaP cells with selectively labeled, fluorescent derivatives of GnRH analogues, including GnRH2 (64). Effective cellular uptake of GnRH2 conjugates were observed in LNCaP cells, which was ascribed to GnRHR1 (64). However, *GnRHR2* is also expressed in these cells (56), so it remains unclear which receptor mediated these effects since GnRHR2 was not examined (64). Of note, uptake of GnRH2 conjugates by LNCaP cells was greater than GnRH1 conjugates or any other cell type tested (human breast, colon, pancreas (64)).

### Other reproductive cancers

#### Cervical

There is a severe lack of information about the potential role of GnRH2 and GnRHR2 in cervical cancer despite the detection of *GnRHR2* mRNA in HeLa cells ( (9, 22); **Table 1**), an important cell line for biomedical and oncology research (90). There is a critical need to better understand the potential function of GnRH2 and GnRHR2 in these cells since cervical cancer is the second most common cancer in women (91).

#### Testis

Although GnRH2 and GnRHR2 have been investigated in the regulation of many different reproductive cancers, there is a gap in our knowledge regarding the potential influence of GnRH2 and GnRHR2 as a therapeutic to treat testicular cancer. This gap is surprising given that GnRH2 and GnRHR2 are both present within the human testis (29, 92) and highly abundant in swine testes (93), an important biomedical model (94). Likewise, *GnRHR2* expression was greatest in marmoset monkey testes compared to 30 other tissues (7). To our knowledge, neither GnRH2 nor GnRHR2 have been evaluated as possible regulators of testicular cancer. Further study is especially important given the recent discovery that a single nucleotide polymorphism in the *GnRH2* gene is associated with both *GnRH2* expression in the testis as well as bone cancer risk (62). Furthermore, *GnRH2* gene polymorphisms were associated with elevated testosterone levels and an increased prostate cancer risk (87).

## Conclusions

**Figure 1:**
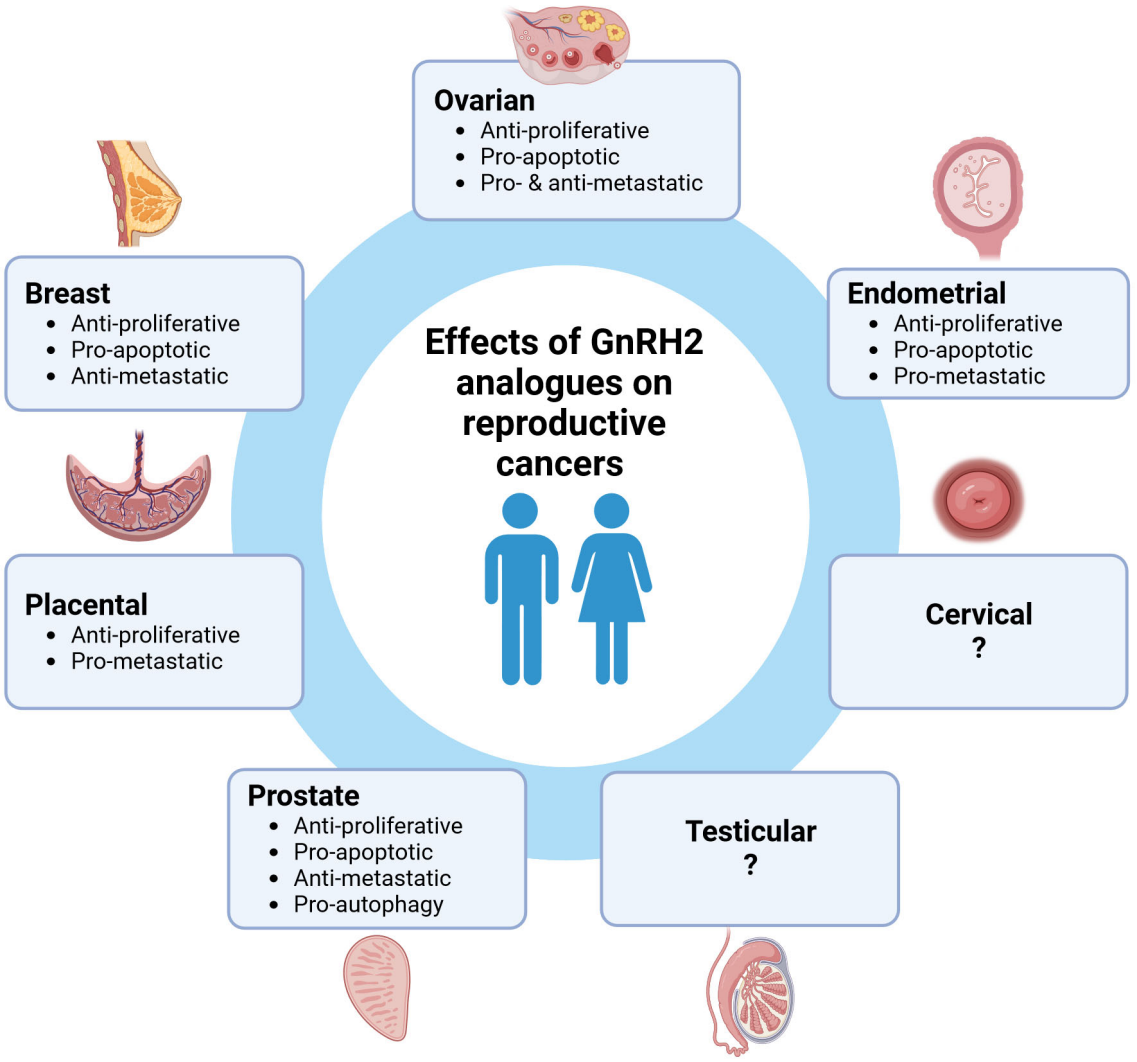
The effect of GnRH2 analogues on human reproductive cancers. Created with BioRender.

GnRH2 and GnRHR2 are expressed in a wide range of human reproductive cancers suggesting an autocrine/paracrine role. Notably, GnRH2 and its analogues mediate potent anti-proliferative and pro-apoptotic activities in many different reproductive cancer cells suggesting an overall inhibitory role (**Figure 1**). However, the metastatic effects of GnRH2 are variable based upon cell type, which remains unresolved. To date, the most widely studied cells have been derived from cancers of the ovary, endometrium, prostate, and breast. However, GnRH2 and/or GnRHR2 are also expressed in other reproductive cancer cells (cervical, placenta), warranting further study. Importantly, the anti-tumor effects of GnRH2 are often more robust than GnRH1, enhancing therapeutic potential. Of concern, the ubiquitous expression of both GnRH2 and GnRHR2 could result in more off-target effects unless GnRH2 analogues could be delivered directly to tumorigenic reproductive tissues (e.g., nanoparticle drug delivery). In addition, further exploration of the connection between methylations/mutations in the *GnRH2* gene with the onset of cancer is essential. Although controversial, the effects of GnRH2 may indeed be mediated via a unique GnRHR2 (e.g., 5-TM). Thus, GnRH2 and GnRHR2 are negative paracrine/autocrine regulators of human reproductive cancers and represent emerging oncological targets.

## Author contributions

AD: Conceptualization, Investigation, Writing – original draft, Writing – review & editing. BW: Writing – review & editing.
